# Affective Neural Responses Sonified through Labeled Correlation Alignment

**DOI:** 10.3390/s23125574

**Published:** 2023-06-14

**Authors:** Andrés Marino Álvarez-Meza, Héctor Fabio Torres-Cardona, Mauricio Orozco-Alzate, Hernán Darío Pérez-Nastar, German Castellanos-Dominguez

**Affiliations:** 1 Signal Processing and Recognition Group, Universidad Nacional de Colombia, Manizales 170003, Colombia; morozcoa@unal.edu.co (M.O.-A.); hdperezn@unal.edu.co (H.D.P.-N.); cgcastellanosd@unal.edu.co (G.C.-D.); 2 Transmedia Research Center, Universidad de Caldas, Manizales 170003, Colombia; hector.torres_c@ucaldas.edu.co

**Keywords:** music-EEG creation, canonical correlation analysis, centered kernel alignment, functional connectivity

## Abstract

Sound synthesis refers to the creation of original acoustic signals with broad applications in artistic innovation, such as music creation for games and videos. Nonetheless, machine learning architectures face numerous challenges when learning musical structures from arbitrary corpora. This issue involves adapting patterns borrowed from other contexts to a concrete composition objective. Using Labeled Correlation Alignment (LCA), we propose an approach to sonify neural responses to affective music-listening data, identifying the brain features that are most congruent with the simultaneously extracted auditory features. For dealing with inter/intra-subject variability, a combination of Phase Locking Value and Gaussian Functional Connectivity is employed. The proposed two-step LCA approach embraces a separate coupling stage of input features to a set of emotion label sets using Centered Kernel Alignment. This step is followed by canonical correlation analysis to select multimodal representations with higher relationships. LCA enables physiological explanation by adding a backward transformation to estimate the matching contribution of each extracted brain neural feature set. Correlation estimates and partition quality represent performance measures. The evaluation uses a Vector Quantized Variational AutoEncoder to create an acoustic envelope from the tested Affective Music-Listening database. Validation results demonstrate the ability of the developed LCA approach to generate low-level music based on neural activity elicited by emotions while maintaining the ability to distinguish between the acoustic outputs.

## 1. Introduction

Sound synthesis refers to the creation of original audio signals by combining procedures that use embedded representations to extract information properties from complex data of different natures. Generated acoustic data have broad applications ranging from artistic innovation to creating adaptive, copyright-free music for games and videos [1], among others. Acoustic representations of music generation are often derived directly from other audio data sources [2]. However, music perception may involve segregating more complex composition structures such as melody, harmony, rhythm, and timbre. Due to the enhanced perception capabilities [3], sound generation has shown considerable potential with Machine Learning (ML) models fed by raw time-domain data, for which architectures are designed to be tightly coupled to the audio representations [4]. However, learning musical styles from arbitrary corpora implies adapting ideas and patterns borrowed from other contexts to a concrete objective. Style learning poses several challenges to ML architectures. Namely, the following issues are reported [5]: capturing/generating music with short- and long-term structures; performing low-level analysis (onset/offset detection, rhythm estimation, harmonic analysis) and high-level analysis (instrument detection, structural segmentation, genre, and mood classification); creating models that possess inherent reasoning to reduce training data requirements; and promoting transparent and objective evaluation methodologies, among others.

The creation of sounds often relies on statistical distributions deduced from training acoustic data or from supplementary media information sources. Reports include the speaker’s voice [6], multimodal audio–visual processing, and multi-instrumental setting [7], text and symbolic transcriptions [8]. Specifically, supplementary data are provided for applying conditioning to deep learning architectures while training [9]. The most common method is adding additional audio data to the input set, taking advantage of vast online music resources. Nevertheless, conditioning strategies for low-level music synthesis may include non-acoustic data used to create audible sounds (also known as sonification [10]), such as speech, images, text, and videos. Moreover, sonification can be used for more unique sources, such as non-empty objects containing fluids [11], mode vibrations of protein and amino acid building blocks [12], and the silent nature of flames [13]. Other sources are biosignals captured from the human body, including electromyography [14] and electrocardiographic data [15]. Even so, electroencephalography (EEG) signals reflect emotions more accurately in real-time than other peripheral neurophysiological data. It also offers more reliable data acquisition hardware with increasing affordability. As an example, EEG-based affective brain–computer interfaces have attracted interest in developing music creation systems [16]. However, the estimation accuracy of induced affective states using EEG signals might be insufficient for applying conditioning to ML architectures [17]. Often, modeling of emotions lacks consistency and is strongly context-dependent [18], not to mention that the brain processes involved in the induction and mediation of affective states by emotionally evocative stimuli are poorly understood due to the difficulty of carefully controlling these types of studies [19].

Several characteristic sets inspired by the human auditory system and physiological findings are used for feature extraction from auditory data. These characteristics offer a broad set of possibilities for automatic descriptions of music signals [20], leveraging the ability to extract acoustic descriptors across a wide dynamic range. Along with acoustic features, spectrograms [21], embeddings and symbolic representations are employed for ML in sound synthesis [22]. In terms of obtaining EEG parameters, there are several limitations. Firstly, the mechanisms evoking emotions are not only related to sound perception and are especially subjective (information focus, cultural impact, musical structure orientation) [23,24]. Next, EEG measures the brain’s electrical activity captured from the scalp, which often contains significant artifacts unrelated to the presented stimulus and caused by other cognitive tasks or reference noises [25]. Due to this, there are no standard methods for extracting features from EEG data within the ML frameworks that have been dedicated to EEG sonification in recent years [26].

Another issue is integrating data from multiple heterogeneous sensors into a low-dimensional representation, learning the joint temporally modulated dependencies from both modalities (audio and EEG) that are assumed to be mutually correlated [27]. Feature reduction and selection are conducted as a first step to handle the large dimensionality of the extracted characteristics and increase their interpretability [28]. As regards the relationship between music stimuli and evoked neural responses, two distinct assessments are reported for music generation: (a) Regression-based approach that directly predicts a real-valued correlation between the coupled sets. (b) Recognition-based approach for coupling the feature modalities through a standard set of categorical labels. The relationship is assessed indirectly by the contribution learned by each training feature assemblage to classifier performance [29]. To date, several multivariate correlation-based methods have been reported to shed light on EEG-based music analysis, including Canonical Correlation Analysis (CCA) that linearly transforms two sets to a domain maximizing their pairwise correlation estimate [30], improved CCA-variant techniques [31], Multifractal Detrended Cross-Correlation Analysis [32], and coupled Nonnegative Tensor Decomposition [33]. Several ML approaches have recently been developed, such as deep CCA that infers the optimum feature mapping [34], and architectures using Convolutional neural networks to compute the similarity between spaces [35], among others. Nonetheless, the performance of these feature alignment strategies described above is adversely affected if the training data is noisy and/or has high variability [36]. Thus, the signal-to-noise ratio of EEG recordings is poor because weak signals are overlaid by intrinsic noise with a much larger amplitude than that generated by biological sources and cause intra-subject and intersubject variability. As a result, feature extraction and feature alignment strategies require multiple repetitions across many runs and trials. However, in stimulus-response paradigms, auditory datasets hold very few trials per individual since participants tend to tire easily or have listening fatigue. Consequently, improving feature alignment strategies to measure the similarity between elicited audio stimuli and evoked EEG responses is still challenging [37].

This work proposes an approach to sonifying neural responses to affective music listening data using the introduced Labeled Correlation Alignment (LCA), which identifies the EEG features that are maximally congruent with the simultaneously extracted auditory features. The proposed two-step LCA approach embraces a separate stage that matches both input features with a set of emotion label sets using Centered Kernel Alignment (CKA). Afterward, Canonical Correlation Analysis (CCA) selects multimodal representations with higher relationships. LCA enables physiological explanation by adding a backward transformation to estimate the matching contribution of each extracted EEG feature set. CCA correlation estimates and partition quality are used as performance measures. To deal with inter/intra-subject variability, we evaluate three feature extraction strategies using Functional Connectivity (FC): the widely used Phase Locking Value, Gaussian Functional Connectivity, and combining both FC measures. The task of discriminating and paying attention to a specific sound source in an auditory environment is complex due to the variability of both the stimuli and the subjects, presenting changes in response in the test subjects and generating challenges in identifying a pattern of activation. In this analysis of neuronal activation in the presence of auditory stimuli, there are studies of auditory attention [38], as well as exploring the relationship between EEG and audio, such as Canonical Correlation Analysis (CCA) [39], for determining the correlation between the spaces. It also finds Neural Networks (NN) [40] to improve the correlation, although still limited since it optimizes the discrimination to represent instead of the final CCA projection [41], in addition to optimizing CCA in pre-training, but not while training the task [42]. In addition to improving the correlation between auditory attention and EEG and discovering the relationship between stimulus-response and BCI, the LCA approach also finds patterns in BCI to generate applications, such as in education and music [43].

Consequently, we identify the EEG features most congruent with evoked auditory data according to each label and present the results accordingly. In order to improve sonification discrimination abilities, we focus on the main aspects. Aspects such as channels, time-windowed dynamics, and bandpass filtering are addressed specifically. Additionally, concrete results of generated discriminative acoustic signals are examined.

The agenda is as follows: Section 2 describes the feature extraction methods, Labeled Correlation Alignment, and the variational autoencoders employed for sonification. Further, Section 3 explains the validated affective music listening database, including the preprocessing procedure and tuning of key parameters for feature extraction. Then, Section 4 summarizes the results in terms of spatial relationship and the effect of time-windowed feature extraction on the LCA performance. Lastly, Section 5 gives critical insights into their supplied performance and addresses some limitations and possibilities of the presented approach.

## 2. Materials and Methods

### 2.1. Extraction of (Audio)Stimulus-(EEG)Responses

A piecewise stationary analysis accounts for the non-stationarity behavior inherent to training data when characterizing the eliciting acoustic stimuli (Y∈R) and brain neural responses (X∈R). Thus, both feature sets (X∈X,Y∈Y) are extracted from $M_{\tau }$ overlapping segments framed by a smooth-time weighting window lasting $\tau_{m}\;\leq \;T$, with $m\in M_{\tau }$, where T∈R is the recording length.

Specifically, a set of time-windowed neural response features, X→X, is extracted from the EEG electrode montage using two functional connectivity metrics (FC), Phase Locking Value (PLV) and Gaussian FC (GFC), estimated on a trial-by-trial basis, respectively, as [44]:

(1a) $$\begin{array}{cc} \Delta \phi_{V}\left(x_{m}^{c},x_{m}^{c^{\prime }}\right)\; & =E\left\{|\exp\left(j\left(\phi_{m}^{c}\left(t\right)-\phi_{m}^{c^{\prime }}\left(t\right)\right)\right)|\;:\;\forall t\in \tau_{m}\right\} \end{array}$$

(1b) $$\begin{array}{cc} \Delta \phi_{G}\left(x_{m}^{c},x_{m}^{c^{\prime }};\sigma_{\phi }\right) & =\exp\left(\frac{-∥x_{m}^{c}-x_{m}^{c^{\prime }}{∥}_{2}^{2}}{2\sigma_{\phi }^{2}}\right) \end{array}$$

where $x_{m}^{c}$ and $x_{m}^{c^{\prime }}$ are the real-valued EEG vectors captured at instant $m\in M_{\tau }$ from the corresponding electrodes $c,c^{\prime }\in N_{C}$; $\phi_{m}^{c}\left(t\right)$ and $\phi_{m}^{c^{\prime }}\left(t\right)$ are the corresponding instantaneous phases $\phi_{m}^{c}\left(t\right)$ and $\phi_{m}^{c^{\prime }}\left(t\right)$, with $c\;\neq \;c^{\prime }$, $N_{C}$ is the number of testing montage channels $\{x_{m}^{c}\in \;\left[x_{m}^{c}\;:\;m\in M\right]\}\in X$, and $\sigma_{\phi }\in R^{+}$ a length scale hyperparameter. Notations ${∥\cdot ∥}_{2}$ and $E\left\{\;\;:\;\forall \nu \right\}$ stand for $\ell_{2}$-norm and expectation operator computed across a variable ν, respectively.

In parallel, a set of time-windowed acoustic features, Y→Y, is extracted under the music assessment and music listening paradigms [45]: Zero-Crossing Rate, Zero-Crossing Rate, High/Low Energy Ratio, Spectral Entropy, Spectral Spread, Spectral Roll-off, Spectral Flatness, Roughness, RMS energy, Broadband Spectral Flux, and Spectral flux for ten octave-wide sub-bands. The extracted acoustic features’ descriptions are detailed in [46,47]. Furthermore, the feature set is completed by the short-time auditory envelopes extracted as in [48].

### 2.2. Two-Step Labeled Correlation Alignment between Audio and EEG Features

The proposed feature alignment procedure between eliciting audio-stimuli and aroused EEG responses consists of two steps: Firstly, the similarity of each feature space to the label set is assessed using Centered Kernel Alignment. This space allows selecting the extracted representations that match the closest. After selecting the labeled CKA representations, Canonical Correlation Analysis is performed to identify audio and EEG features that are maximally congruent in terms of estimated correlation coefficients.

#### 2.2.1. Supervised CKA-Based Selection of Features

Sonification feature sets must be selected to create music following brain patterns but according to distinct emotional conditions. Hence, the alignment is performed separately between each feature set, $\Xi \in \;\{X\in R^{N_{R}\times P}$, $Y\in R^{N_{R}\times Q}\}$ being *P* and *Q* the number of EEG and Audio features ($N_{R}$ is the number of trials), to the provided labels, noted as Λ∈Z, employing the CKA algorithm that includes an additional transformation to estimate the contribution of every input representation. To be specific, we use the supervised empirical estimate of CKA derived in [49], as follows:

(2) $$\begin{array}{cc} {w_{\Xi }}^{*} & =\underset{W_{\Xi }}{\arg\max}\frac{{\langle {\bar{K}}_{\Xi }\left(W_{\Xi }\right),{\bar{K}}_{\Lambda }\rangle }_{F}}{\left|\right|{\bar{K}}_{\Xi }\left(W_{\Xi }\right){\left|\right|}_{F}\left|\right|{\bar{K}}_{\Lambda }{\left|\right|}_{F}}; \end{array}$$

where notation $\left|\right|\cdot {\left|\right|}_{F}$ stands for Frobenius norm, $\bar{K}\in R^{N_{R}\times N_{R}}$ is the centered kernel matrix estimated as $\bar{K}=\tilde{I}K\tilde{I}$, $K\in R^{N_{R}\times N_{R}}$ is the kernel matrix, $\tilde{I}=I-1^{⊤}1/N_{R}$ is the empirical centering matrix computed across the trial set that holds $N_{R}$, and $I\in R^{N_{R}\times N_{R}}$ is the identity matrix, $1\in R^{N_{R}}$ is the all-ones vector; and $K_{\Xi }\in R^{N_{R}\times N_{R}}$ and $K_{\Lambda }\in R^{N_{R}\times N_{R}}$ are the kernel matrices that match each extracted feature set to the labels, respectively.

The kernel matrix elements, $\xi ,\xi^{\prime }\in \Xi$, are computed on a trial-by-trial basis, respectively, as follows:

(3a) $$\begin{array}{cc} \kappa_{\Xi }\left(\xi ,\xi^{\prime };W_{\xi }\right) & =\exp\left(-\left({\left(\xi -\xi^{\prime }\right)}^{⊤}W_{\xi }^{⊤}W_{\xi }\left(\xi -\xi^{\prime }\right)\right)/2\right), \end{array}$$

(3b) $$\begin{array}{cc} \kappa_{\Lambda }\left(\lambda ,\lambda^{\prime }\right) & =\delta \left(\lambda ,\lambda^{\prime }\right),\;\lambda ,\lambda^{\prime }\in \Lambda \end{array}$$

where $W_{\xi }$ is the matrix linearly transforming the selected $\tilde{\xi }$ and input ξ sets in the form $\tilde{\xi }=\xi W_{\xi }$, with $\tilde{\xi }\in \left\{\tilde{X}\in R^{N_{R}\times P},\tilde{Y}\in R^{N_{R}\times Q}\right\}$, being $W_{\xi }W_{\xi }^{⊤}$ the corresponding inverse covariance matrix of the multivariate Gaussian function as in Equation (3a).

A Gaussian function is used as the first kernel $\kappa_{\Xi }\left(,\right)\in R^{+}$ in Equation (3a), to assess the pairwise similarity between aligned features due to its universal approximation properties and tractability [50]. The second kernel includes the delta operator δ(·,·) in Equation (3b) suitable for dealing with categorical label values.

#### 2.2.2. CCA-Based Analysis of Multimodal Features

This unsupervised statistical technique aims to assess the pairwise linear relationship between the multivariate projected feature sets $\tilde{\Xi }=\;\left\{\tilde{X},\tilde{Y}\right\}$ obtained by supervised CKA-based selection and described in different coordinate systems (EEG and Audio). To this end, both representation sets are mapped into a common latent subspace to become maximally congruent. Namely, the correlation between the EEG and auditory features is maximized across all $N_{R}$ trials within a quadratic framework constrained to a single-dimensionality latent subspace, as below [51]:

(4a) $$\begin{array}{cc} {\hat{\alpha }}_{\tilde{X}},{\hat{\alpha }}_{\tilde{Y}} & =\underset{\alpha_{\tilde{X}},\alpha_{\tilde{Y}}}{\arg\max}\alpha_{\tilde{X}}^{⊤}\Sigma_{\tilde{X}\tilde{Y}}\alpha_{\tilde{Y}} \end{array}$$

(4b) $$\begin{array}{cc} s.t.: & \alpha_{\tilde{X}}^{⊤}\Sigma_{\tilde{X}\tilde{X}}\alpha_{\tilde{X}}=1,\;\alpha_{\tilde{X}}\in R^{P} \end{array}$$

(4c) $$\begin{array}{cc} \; & \alpha_{\tilde{Y}}^{⊤}\Sigma_{\tilde{Y}\tilde{Y}}\alpha_{\tilde{Y}}=1,\;\alpha_{\tilde{Y}}\in R^{Q} \end{array}$$

where $\Sigma_{\tilde{X}\tilde{X}}\in R^{P\times P}$, $\Sigma_{\tilde{Y}\tilde{Y}}\in R^{Q\times Q}$, and $\Sigma_{\tilde{X}\tilde{Y}}={\tilde{X}}^{⊤}\tilde{Y}\in R^{P\times Q}$.

### 2.3. Sonification via Vector Quantized Variational AutoEncoders

The feed-forward encoder and decoder network converts an input time-series $\xi =\;[\xi_{t}\;:\;\forall t]$, with ξ∈Ξ, into a coded form of a discrete finite set (or tokens), $z\in \{z_{s}\;:\;\forall s\in S\}$, having each element of size *K*. To this end, a latent representation $h_{s}=\theta_{E}\left(\xi \right)$ (with $H\in \{h_{s}\}$) is encoded to be further element-wise quantized according to the vector-quantized codebook $\left\{e_{k}\;:\;\forall k\right\}$. The VQ-VAE model noted as μ(ξ) is then trained using the minimizing framework, as below [52]:

(5) $$\begin{array}{c} \mu \left(\xi \right):\min E\left\{{∥\xi_{t}-\theta_{D}\left(e_{z,t}\right)∥}_{2}^{2}:\forall t\right\}\\ \;+E\left\{{∥\theta_{SG}\left(h_{s}\right)-e_{z,s}∥}_{2}^{2}:\forall k\right\}+\beta E\left\{{∥h_{s}-\theta_{SG}\left(e_{z,s}\right)∥}_{2}^{2}:\forall k\right\} \end{array}$$

where the first term is the reconstruction loss that penalizes for the distance between input ξ and decoded output $\tilde{\xi }=\theta_{D}\left(\cdot \right)$, the second term penalizes for the distance between each encoding value of *H* and their nearest neighbors $e_{z}$ in the codebook, and the third term prevents the encoding from strong fluctuations, ruling the weight β∈R[0,1]. In addition, notation $\theta_{SG}\left(\cdot \right)$ stands for the stop-gradient operation, which passes zero gradients during backpropagation.

Generally speaking, the coding model trained by one auditory signal set ξ∈Ξ can be applied to the generation of acoustic data when feeding to the encoder signals of different nature, $\xi^{\prime }\in \Xi$, provided their homogeneity is assumed. This model is referred to as $\mu (\xi |\xi^{\prime })$. In light of this, we suggest that the following conditions be met:

– The VQ-VAE coder includes a parametric spectrum estimation based on regressive generative models fitted on latent representations [53]. Therefore, both sets of signals ($\xi ,\xi^{\prime }$) must have similar spectral content, at the very least, in terms of their spectral bandwidth. That is,
  (6) $$\Delta F_{\xi }≃\Delta F_{\xi^{\prime }}$$
– In regression models, both discretized signal representations must be extracted using similar recording intervals and time windows to perform the numerical derivative routines. Furthermore, the VQ-VAE coder demands input representations of fixed dimensions. Hence, the arrangements extracted from ξ and $\xi^{\prime }$ must be of similar dimensions.

## 3. Experimental Setup

**Figure 1:**
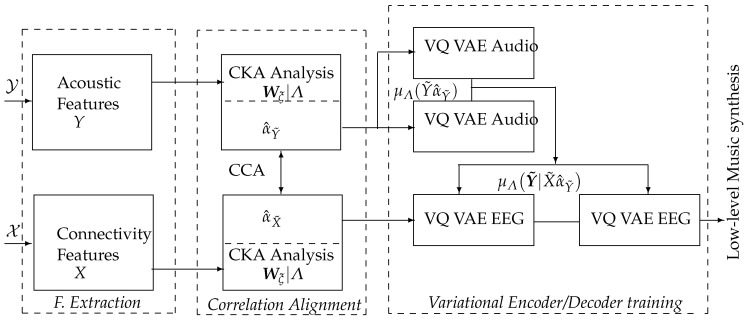
Proposed model architecture. The inputs are the neural activity (X) and auditory (Y) data acquired under the Stimulus-Response Paradigm, and the output is the set of acoustic envelopes for creating labeled low-level music content.

We propose a method for sonifying neural responses to labeled affective music listening using auditory and electroencephalographic features that are maximally congruent with the label set. The method is evaluated to create music within the stimulus-response paradigm using a scheme that encompasses the following stages (see Figure 1):

(i) Preprocessing and extracting time-windowed representations: Estimating acoustic features from music data modulating emotions, and Functional Connectivity measures from evoked EEG neural responses. Three strategies for FC extraction are considered: Phase Locking Value, Gaussian Functional Connectivity, and their combination. Different time windows are evaluated for feature extraction from neural brain responses as the conditioning content is devoted to low-level music generation.

(ii) Labeled Correlation Alignment to identify the EEG features that are maximally congruent with the stimulating auditory data by each emotion. To preserve the interpretability of selected arrangements, this stage is performed in a two-step procedure: separate CKA matching between audio and EEG data with the labels, followed by CCA analysis of the selected feature sets.

The contribution of electrodes and bandpass-filtered, time-windowed dynamics to Labeled Correlation Alignment is examined. The subject’s influence on overall performance is also considered.

(iii) Labeled audio conditioning content was generated using selected brain neural responses to feed a Vector Quantized Variational AutoEncoder.

We assess the relationship between the neural responses captured and the auditory data in terms of their correlation estimated by CCA as a performance measure. Namely, the higher the *r*-squared coefficient, the more related the brain responses to auditory stimuli. The leave-one-out cross-validation strategy is applied (more precisely, leave-one-subject-out) to compute the confidence of CCA correlation estimates, as carried out in [54]. The discrimination ability of the labeled correlation alignment is also evaluated through the clustering coefficient, $\gamma \in R^{+}$, that is the partition quality of the CCA correlation values, computed as:

$$\gamma =\left(\frac{\xi_{1}-\xi_{0}}{\max_{i}\xi_{i}}+E\left\{{\left(\xi_{n}-\bar{\xi }\right)}^{2}\;:\;\forall n\in N_{R}\right\}\right),\;\xi_{i}\in \Xi$$

where $\xi_{0}$ is the mean distance between a sample and all other points in the same group, $\xi_{1}$ is the mean distance between a sample and all other points in the closest group, $\xi_{n}$ is the number of samples within the data set, $\bar{\xi }$ is the center of a group, where the squared distance of each sample to the center of each group is calculated [55]. This clustering measure calculates a trade-off between inter-class (first term) and intra-class variability (second term). Consequently, the larger the value of γ, the more different the labeled partitions of the extracted features will be.

### 3.1. Affective Music Listening Database

The data (publicly available at https://openneuro.org/datasets/ds002721/versions/1.0.2) (accessed on 1 April 2023) were collected by a total of $N_{S}=31$ individuals. The test paradigm consisted of six runs, capturing brain neural responses divided into two parts: baseline resting recordings were measured while the participants were sitting still and looking at the screen for 300 s (first and last run); four intervening runs (that is, $N_{R}=40$ trials per subject), each with ten individual trials. During a single trial, a fixation cross was presented until 15 [s] had passed. A randomly selected musical clip was played for T=12 s after the fixation cross appeared. The participants were given a short break after listening to musical stimuli, followed by eight questions in random order to rate the music on a scale (1–9) of induced pleasantness, energy, tension, anger, fear, happiness, sadness, and sadness tenderness. Each participant had 2–4 s between answering the last question and the subsequent fixation cross in the inter-trial intervals.

**Figure 2:**
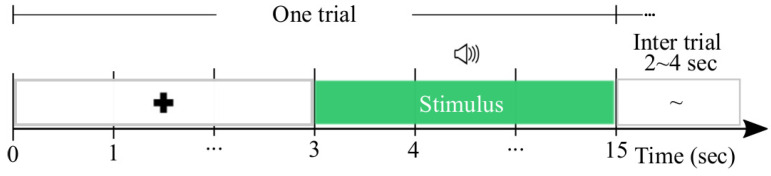
The experimental paradigm used by affective music listening-database.

For each subject, the signal set was recorded from 19 channels according to 10–20 electrode placement (Fp1, Fp2, F7, F3, Fz, F4, F8, T3, C3, Cz, C4, T4, T5, P3, Pz, P4, T6, O1, and O2), and each recording lasting 15 s was sampled at a rate of 1000 Hz, submitted in Figure 2. The music stimuli examined how music modulates emotions and contained 110 excerpts from scores covering a wide range of emotional responses, as detailed in [56]. It is worth noting that the auditory data are labeled according to the two-dimensional arousal-valence plane since affective states may be characterized as a consciously accessible condition that combines arousal (activated-deactivated) and valence (pleasure-displeasure), resulting in the following four labeled partitions ($N_{L}=4$) [57]: High Arousal Positive Valence states (HAPV), High Arousal Negative Valence (HANV), Low Arousal Negative Valence (LANV), and Low Arousal Positive Valence (LAPV).

### 3.2. Preprocessing and Feature Extraction

#### 3.2.1. Time-Windowed Representations of Brain Neural Responses

Preprocessing EEG data consists of the following procedures:

(i) High-pass filtering of the raw EEG channel set was performed with a relatively high cutoff frequency to remove linear trends in all $N_{C}$ electrodes. To this end, a zero-phase 3rd-order Butterworth filter was employed to bandpass the raw signal within [1–45] Hz. Further, the FC feature sets were extracted within a bandwidth $f\in N_{B}$, with $N_{B}\;=\;\left\lfloor F_{s}/2\right\rfloor$, where $F_{s}\in R^{+}$ represents the EEG sampling frequency. The bandwidths were selected to cover physiological rhythms, which are influential in music appraisal within EEG paradigms, as reported in previous studies [58]. Namely:

θ [4–8] Hz, α [8–12] Hz, and β [12–30] Hz; (ii) Artifact removal was achieved for the occipital electrodes (associated with motor control) that may be highly active because of the visual perception of sound stimuli after target presentation [59]. Another factor contributing to poor occipital signals might be insufficient electrode contact [60]. In this regard, the impedance had outlier values of (>100 kΩ) in three subjects. Therefore, both channels (O1, O2) were ignored in the following. (iii) Re-reference to the common-average electrical activity measured across all scalp channels. (iv) Resampling of EEG responses, partitioned by trials, using the onset of each music stimulus as a fiducial mark, and further downsampling at the sampling rate of 80 Hz. (v) Lastly, the piecewise stationary analysis of EEG and auditory data was carried out over a set of the time segments (having testing values [12,6,3,1.5,0.75, and 0.375] s), windowed by a smooth-time weighting function (namely, Hann window) with 50% overlap.

Further, the FC features are extracted according to Equations (1a) and (1b), where the kernel bandwidth parameter of GFC is optimized to reduce the probability density function variability of the observed data $p(X|\sigma_{\phi })$, that is, as detailed in [61]:

$${\tilde{\sigma }}_{\phi }=\arg\max\mathrm{var}\left\{p\left(X|\sigma_{\phi }\right)\right\}$$

As a result, we extract one real-valued FC matrix sizing $N_{\phi }\times N_{\phi }$, in a single trail-basis at instant τ, for each evaluated FC measure and subject.

The FC matrix is vectorized to have a vector dimension $N_{\mathrm{FC}}=N_{\phi }\left(N_{\phi }-1\right)/2$. Accordingly, the feature vector derived from individuals, $N_{S}$, across all trials, $N_{R}$, includes dimension $N_{\tilde{X}}^{\lambda }=N_{FC}\times N_{\tau }\times N_{T}\times N_{S}\times N_{L}$, extracted from each emotion label λ for purposes of validating the supervised feature alignment. Note that the extracted EEG feature arrangement doubles in size when both FC measures are concatenated.

#### 3.2.2. Time-Windowed Representations of Eliciting Audio Stimuli

Regarding auditory stimuli, all recordings were sampled at 44,100 Hz and then segmented into $N_{\tau }$ sliding windows with 50% overlap. Moreover, the sampled data are smooth by squaring and applying a convolution with a square window. As a way to fulfill the condition in Equation (6), stimuli data are further downsampled to 64 Hz with cubic root compression. In order to match the dimension of the EEG training set, the acoustic set is also fixed to a similar size, that is, $\dim\left(\tilde{Y}\right)\;∼\;\dim\left(\tilde{X}\right)$. Therefore, within each τ, we extract the first PCA component from each of the 20 acoustic features described above [62]. The array is completed with $N_{\phi }-1$ samples of the acoustic envelope. So, we extract $N_{\tau }\left(20+N_{\phi }-1\right)$ acoustic features within each *T* to be fed into the next alignment procedure.

## 4. Results

Here, we present the results by selecting the EEG features most congruent with the evoked auditory data according to each label. We focus on the main aspects to improve the sonification process’s discrimination abilities. Specifically, we address the influence of channels, time-windowed dynamics, and bandpass filtering on neural responses. Concrete outcomes of generated discriminative acoustic signals are also analyzed.

### 4.1. Electrode Contribution to Labeled Correlation Alignment

**Figure 3:**
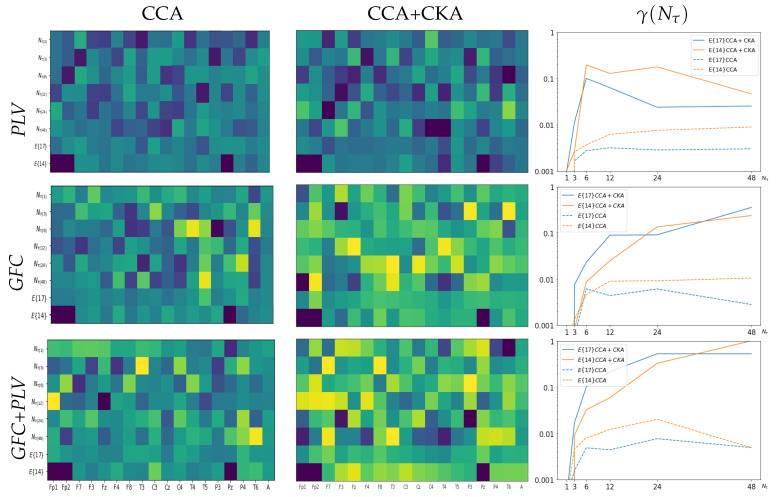
The electrode contribution of *r*-squared values and clustering coefficients γ (right column) obtained by the validated strategies of feature extraction: PLV (Top row), GFC (middle row), and their combination (Bottom row). Notations $E\left\{17\right\}$ stands for all EEG channel signals (i.e., $N_{C}=17$) excluding O1,O2 while $E\left\{14\right\}$ denotes without frontoparietal (Fp1, Fp2) and Midline Parietal (Pz) electrodes ($N_{C}=14$), respectively. The horizontal axis stands for each electrode according to the standard 10–20 system. In the right column, the horizontal axis denotes each considered time-windowed set, $N_{C}$.

In the beginning, we consider the spatial relevance of each electrode in the scalp EEG montage in terms of the relationship reached by LCA between the features extracted from neural responses and acoustic stimuli. Figure 3 shows the *r*-squared values assessed by CCA after applying CKA matching (middle column), which are displayed at each validated set of window intervals, $N_{\tau }$. The correlation estimates are averaged across the label set for a generalized interpretation. As can be seen from the plotted heatmaps, the correlation range varies and spreads differently over the scalp electrodes depending on the evaluated feature extraction method. This fact can be seen in the top heatmap revealing that PLV obtains the lowest estimates between [0.05–0.59], with very few electrodes having a detectable contribution. In contrast, GFC extends the correlation interval to [0.05–0.73] (middle plot). At the same time, combining both measures results in correlation values [0.10–0.74] (bottom plot), suggesting that either strategy of improved FC extraction leads to apparent brain regions being coupled to the acoustic stimuli.

Afterward, we evaluate the influence of each channel by averaging its correlation performance across all tested window intervals, as displayed in the matrix row for the whole EEG montage (noted as $E\left\{17\right\}$). It is worth noting that several electrodes tend to zero-value their contribution regardless of the extraction method employed. A particular focus is placed on electrodes that have been reported to be susceptible to artifacts during data acquisition of music listening paradigms, specifically, the ones associated with brain neural activity in the frontal cortex [63]. Thus, the bottom row (noted as $E\left\{14\right\}$) presents the averaged *r*-squared values and shows that the correlation may increase when removing Fp1, Fp2, and Pz electrodes.

The next aspect of consideration is evaluating the discrimination ability of the selected features using the clustering coefficient γ. As displayed in the right column of Figure 3, the partition separability of features extracted by PLV (see top plot) is modest due to the low assessed *r*-squared values. In the case of GFC, the partitions between extracted EEG features differ more pronouncedly. At the same time, the combination of GFC and PLV provides the most accurate separable clustering performance across the tested values of the time window τ. Observed behavior remains for each electrode arrangement evaluated: $N_{C}=17$ (blue line) or $N_{C}=14$ (orange line). For comparison, we assess the discrimination ability of each feature selection procedure after conducting just a single CCA step that achieves a significantly lower correlation (see left column) than the values attained by incorporating the supervised CKA step previously (middle column). A comparison of the heatmaps shows that a single CCA step results in lower values of γ (dashed lines) regardless of the extraction method used, indicating the increased association between neural responses and acoustic stimuli achieved through LCA.

**Figure 4:**
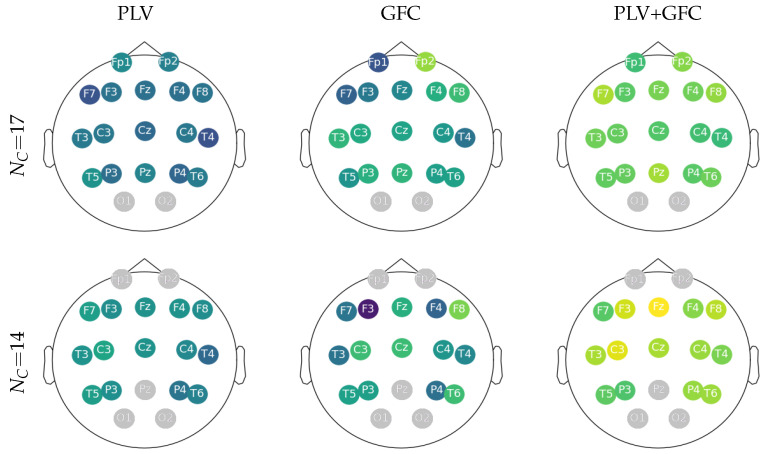
Topoplots reconstructed from LCA according to the estimated electrode relationship with the evoking auditory data. The channels affected by artifacts in gray are removed from the coupling analysis.

Lastly, for purposes of physiological interpretability, Figure 4 displays the topoplots reconstructed from the FC feature sets according to the correlation with the evoking auditory data performed by LCA. As seen in the left column, PLV delivers weak values of *r*-squared that are evenly distributed over the scalp. On the other hand, GFC increases both lobes’ contribution (see central column). This influence is further accentuated by combining GFC with PLV, giving rise to electrodes with powerful relevance (right column) and thus increasing their relevance in the following sonification stages. Note that correlation assessments focus more on the frontal and central lobes (painted yellow) when artifact-affected electrodes are removed.

### 4.2. Correlation Estimation for Time-Windowed Bandpass Feature Sets

**Figure 5:**
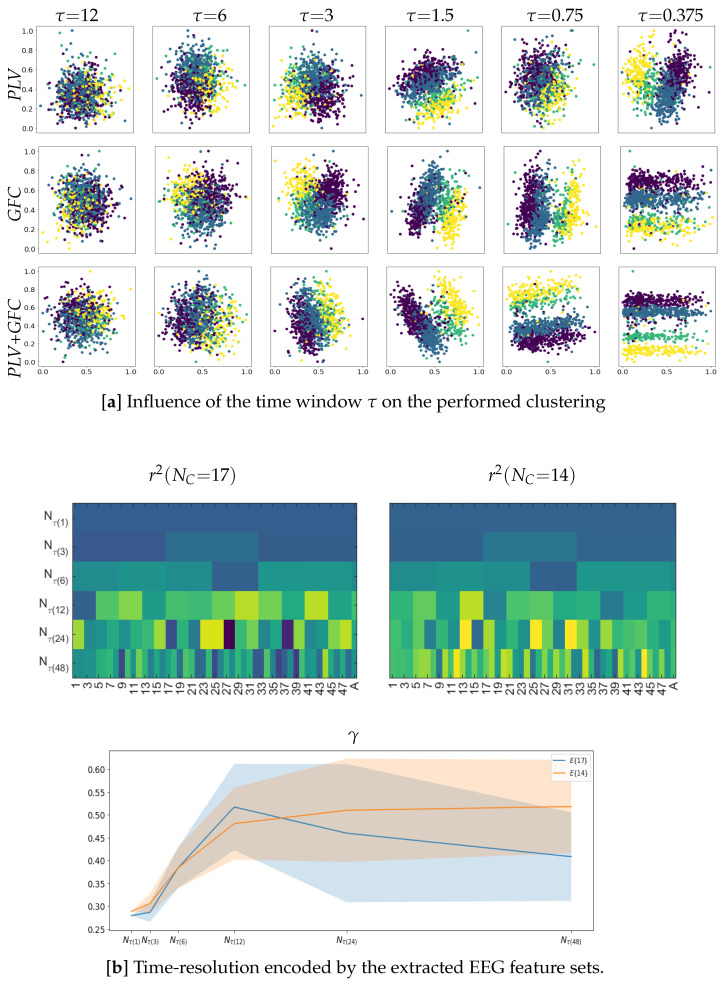
Effect of time-windowed dynamics on the estimated values of *r*-squared. (**a**) Quality of clustering between labeled affective neural responses depending on the time window length $\tau_{m}$ measured in *s*. Outcomes are presented just for the removal channel configuration $N_{C}=14$ since it enhances the γ values. (**b**) Dynamic resolution of neural responses encoded by the extracted feature sets. The influence of both channel removal configurations is evaluated. Of note, only the method combining PLV + GFC is evaluated, and clustering is performed over the reduced set of EEG features using Principal Component Analysis separately for each affective label.

Here, we investigate the effect of applying time-windowed feature extraction on LCA performance and, in particular, how distinct the EEG responses remain over time since changing dynamics can play a significant role in music creation. To illustrate this aspect, the upper plot of Figure 5 unfolds the time-varying clustering coefficient at different windows performed by each extraction method in the previous section (see Figure 3). The pictured scatter plots indicate that the labeled EEG feature partitions become distinguishable when fixed to a window narrower than τ≤3 s, meaning that the captured affective neural responses can be more separable regardless of the FC metric used. From this length value down, the narrower the overlapping time segment of feature extraction, the more apparent the neural dynamics become. Note that the labeled partitions of the extracted EEG dynamics differ and are more pronounced in GFC (middle row of the top plot) than in PLV (upper row). However, combining GFC and PLV provides the best group separation (lower row).

Next, we analyze the time evolution of LCA to determine the dynamic resolution of neural responses encoded by the extracted feature sets over time, but only for the best strategy of FC representation (that is, the combination of PLV plus GFC). The lower plot in Figure 5 presents the obtained *r*-squared values and reveals that the dynamics extracted at short lengths of τ are weak because of very wide τ≥3 s, resulting in intervals with almost zero-valued correlation. Comparatively, extracted features at τ≤3 s become stronger and has fluctuations over time (left plot of bottom row). Note that implementing the channel removal strategy (middle plot) improves this behavior. Further, the right plot shows the mean estimate of changes in the time-varying dynamic resolution computed as the difference between neighboring correlation values, revealing that the separability of affective labels tends to decrease as τ shortens. This effect may however be reduced with a proper channel selection, as mentioned previously.

**Figure 6:**
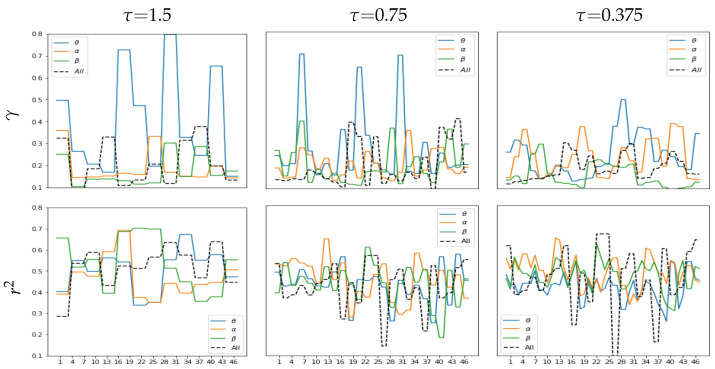
Performance variability over time conditioned by the wavebands θ, α and β. Clustering coefficient (top row) and correlation (bottom row) are estimated at short lengths of window τ using the FC extraction combining PLV + GFC.

Another thing we discuss is the bandpass filtered feature extraction following brain oscillations as a valuable musical property. Figure 6 presents the values of *r*-squared and γ calculated by combining PLV plus GFC and extracted at different time windows for three brain oscillations evaluated (i.e., θ,α,β). Filtering the lowest band (θ waveform painted in blue line) causes more smoothing changes in the obtained time-varying dynamic resolution than the baseline signal holding all waveforms (black line). In contrast, extraction of the higher frequency rhythms (α—orange, β—green) speeds up the time-varying changes in estimated correlation values (bottom row). However, rapid changes in *r*-squared imply that discriminability between affective neural responses fluctuates over time (top row).

**Figure 7:**
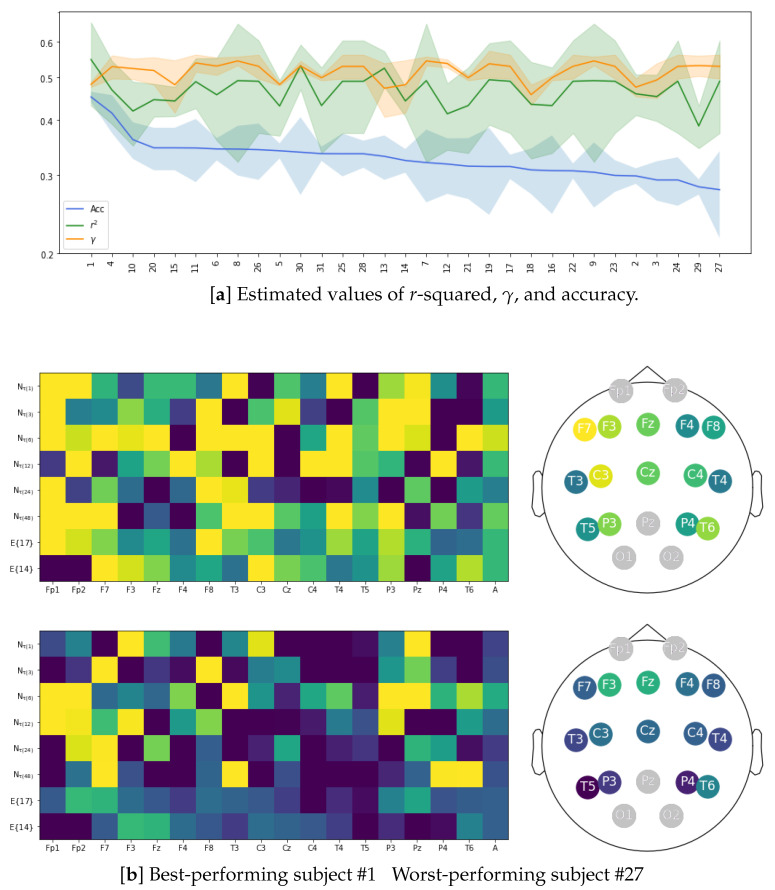
Overall subject performance of LCA. (**a**) Estimated values of *r*-squared, γ, and classifier accuracy. (**b**) *r*-squared heatmaps of electrode contribution and their reconstructed topoplots for subjects #1 and #27. Outcomes are presented for both removal channel configurations $E\left\{17\right\}$ and $E\left\{14\right\}$ using the FC extraction combining PLV + GFC.

To check for uniformity of the group of test subjects, we present in Figure 7 (top plot) the performance of LCA implementation, achieved individually across the channel set and at the considered time windows, which was used for feature extraction based on the combination of PLV plus GFC. In the case of *r*-squared estimation (green line), there is an appreciable discrepancy in mean and variance values among subjects. Furthermore, a few individuals with a high standard deviation may indicate that their elicited neural responses are far from typical in the subject set. In light of the discrimination ability that motivates the LCA algorithm, we compute the classification of affective feature sets using a GraphCNN framework, similar to the approach presented in [64]. The blue line depicts the calculated classifier accuracy values (mean and standard deviation). In order to provide a better understanding, all subjects are ranked in decreasing order of their achieved mean value, showing a large gap between the best and lowest performers. To illustrate this point, we compute the heatmap of electrode contribution from the *r*-squared assessments carried out by both subjects along with the corresponding reconstructed neural activity topoplots. As can be seen in the bottom plot, the best-performing subject (labeled as # 1) reaches a robust relationship between auditory and EEG responses with marked brain zones of activation. Moreover, enhanced performance occurs even within the broadest time window. On the contrary, the worst-performing subject (labeled as # 27) achieves a very scarce correlation heatmap, suggesting a poor contribution from the central brain zone, which is assumed to be important in the Affective Music Listening paradigm.

### 4.3. Generation of Affective Acoustic Envelopes

In the last part of the evaluation, we investigate the ability to create music conditioning content using brain neural activity selected by LAC. Specifically, the VQ-VAE framework in Equation (5) is trained with affective music stimuli, $\tilde{Y}$, and then applied to create auditory data by feeding the autoencoder with the most similar representation of aroused brain neural responses, $\tilde{X}$, i.e., using the model $\mu_{\Lambda }\left(\tilde{Y}\;|\;\tilde{X}\right)$. Due to the highly complex music structure encoded, additional settings are required. Only the acoustic envelope is provided to the encoder as auditory training feature data, without any weighting filter (That is, $W_{\tilde{Y}}=1$), omitting the remaining acoustic features and smoothed to decrease abrupt changes. When providing EEG data to feed the encoder input, the feature sets have an additional dimension to represent neural activity’s spatial contribution. We map the EEG feature matrix into a vector representation by adding one convolutional layer to the VQ-VAE input to reduce dimension.

**Figure 8:**
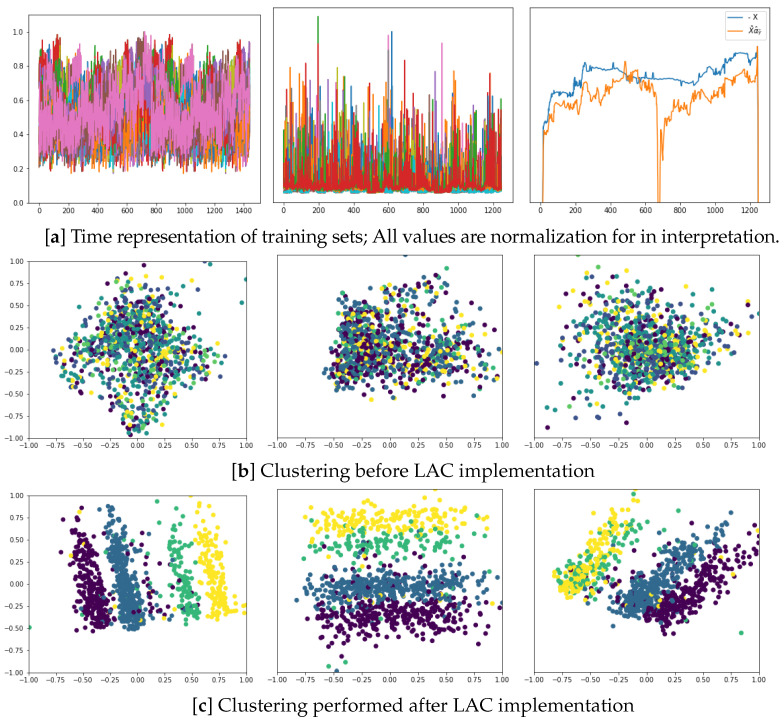
Sonification via VQ-VAE based on the features extracted by LAC. (**a**) Time representation of training sets: Input EEG recordings (left plot), extracted FC measures (central plot), and output acoustic envelopes (right plot); (**b**) Clustering before LAC implementation; (**c**) Clustering performed after LAC implementation. The illustration is given for the arisen EEG responses (left column), FC measures (central column), and created acoustic envelopes (right column).

In the top row, the left plot of Figure 8 illustrates an example of a multichannel EEG response, followed by the extracted FC arrangement (middle plot) and applied to the Labeled Correlation Alignment, estimating the correlation assessments for feeding to the encoder. An example of the generated acoustic envelope in the output is then presented (right plot), reconstructed using VQ-VAE. The right plot illustrates how the envelope resulting from the training model $\mu_{\Lambda }\left(\tilde{Y}|\tilde{X}{\hat{\alpha }}_{\tilde{X}}\right)$ is smooth enough (orange line). As a comparison, we show the acoustic output produced when encoding the raw EEG set directly (i.e., $\mu_{\Lambda }\left(\tilde{Y}|\tilde{X}\right)$ ), showing more increased variability and abrupt changes (blue line), which tend to degrade the overall quality of the created music. In the middle row, we show the clustering results obtained by the sets employed for training: input EEG envelopes (left plot), input FC features (center plot), and generated acoustic envelopes under the model $\mu_{\Lambda }\left(\tilde{Y}|\tilde{X}{\hat{\alpha }}_{\tilde{X}}\right)$ (right plot), which show a low discriminant between affective labeled sets. On the other hand, the Labeled Correlation Alignment makes the compared input training sets distinctive.

## 5. Discussion and Concluding Remarks

This work proposes an approach to sonifying neural responses to affective music listening data. Based on a set of emotions provided, the Labeled Correlation Alignment identifies EEG features most compatible with auditory data. To this end, LCA embraces two steps: Supervised CKA-based feature selection followed by CCA-based analysis. The validated results from the tested real-world data set demonstrate the developed LCA approach’s ability to create low-level music content based on neural activity elicited by the considered emotions, maintaining the ability to discriminate between the produced acoustic envelopes.

Still, after the evaluation stage, the following points are worth noting:

*Feature extraction.* Gaussian Functional Connectivity, characterizing the elicited brain activity, enhances the relationship assessment compared to the widely used Phase Locking Value alone. However, both FC measures’ combinations better associate the neural responses triggered by coupled acoustic stimuli. This result suggests that the correlation may benefit from including kernel-based FC to deal with inter-/intra-subject variability. Nevertheless, the validation shows that the electrodes mostly affected by artifacts must be adequately removed to improve the EEG feature extraction step. This aspect raises the need to consider including other connectivity measures such as Phase-Amplitude Coupling and entropy-based FC representations, also used in music appraisal paradigms.

Regarding auditory representations, the validation results demonstrate that short-time acoustic envelopes can complete the widely used methods of acoustic feature extraction. Moreover, to properly estimate the intrinsic latent stochastic models, these envelopes, coding relationships between neighboring samples, are only fed into the variational encoder network that generates low-level music synthesis. Despite this, more elaborate representations, such as the Musical Instrument Digital Interface format, may be required when encoding music structures of higher complexity.

*Labeled Correlation Alignment*. We introduce the two-step procedure to associate multimodal features aligned with the label set, motivated by the fact that a single step of Canonical Correlation Analysis tends to result in cases of a weak association between coupled representation spaces. Additionally, this method for exploring relationships does not benefit from label set information, resulting in poor discrimination between affective responses. Hence, before Canonical Correlation Analysis identifies highly congruent multimodal features, Centered Kernel Alignment is performed to select the most relevant representations based on the affective labels.

Further physiological explanation of LCA results is possible by adding a backward transformation within CKA to estimate the contribution of each extracted feature set. In particular, the proposed LCA between the elicited audio-stimuli and aroused EEG responses enables interpretation of the following aspects: (a) Electrode contribution shows the correlation estimates focus more on the frontal and central lobes, increasing their relevance in the sonification stage. (b) The contribution, obtained by short-time dynamics, indicates that for narrow windows (τ≤3 s) LCA can deliver affective neural responses that are still separable. Furthermore, the bandpass-filtered feature extraction based on brain oscillations may smooth or speed up EEG dynamics. However, discriminability between affective neural responses can reduce. (c) Influence of participants. A noticeable difference exists between the subject performing best and the one with the lowest accuracy in the assessed correlation.

From the information above, several aspects can be considered for enhancing the association between multimodal features, such as group-level analysis to search for joint contributions across individuals and correlation methods that search for optimized projections, for instance, using deepCCA [65].

*Generation of low-level music content*. Another finding is that the employed variational autoencoder can generate distinctive acoustic envelopes from EEG representations selected by LCA. However, the encoder network uses a discrete latent representation paired with an autoregressive decoder specially designed for high-quality videos, music, and speech. Hence, more efforts are needed to approach discrete neural representation with the predictive VQ-VAE model.

In the future, the authors intend to develop a framework based on variational encoder networks, for which brain neural data can directly affect the latent stochastic representations and regression models involved, according to the estimated relationship between the coupled spaces. More databases, built according to paradigms other than stimulus–response, will also be validated to deal with information shortages.

## Data Availability

The EEG datasets (https://openneuro.org/datasets/ds002721/versions/1.0.2) for this study can be found in the (Music-EEG-Activity) (https://github.com/FrankYesid/Music-EEG-Activity.git) (accessed on 1 April 2023).
